# Increased Intracranial Pressure on a Patient with Neuromyelitis Optica Spectrum Disorder

**DOI:** 10.22088/cjim.12.0.435

**Published:** 2021

**Authors:** Seyed Mohammad Forouzannia, Pourya Yarahmadi, Mohammad Alirezaei, Nasim Rezaeimanesh, Abdorreza Naser Moghadasi

**Affiliations:** 1 Multiple Sclerosis Research Center; Neuroscience institute; Tehran University of Medical Sciences; Tehran; Iran

**Keywords:** Neuromyelitis optica spectrum disorder, High intra cranial pressure, Anti- aquaporin antibody

## Abstract

**Background:**

Neuromyelitis optica spectrum disorder (NMOSD) is an autoimmune astrocytopathic disease affecting central nervous system (CNS). CSF pressure in these patients is usually normal.

**Case Presentation:**

A 30-year-old woman was admitted with complaints of headache and lower limbs paresis. Lumbar puncture (LP) and magnetic resonance imaging were performed for the patient. Opening pressure was 42 cm H2O in the first LP. According to the clinical evidences, imaging, and the patient's positive aquaporin-4 antibody, the diagnosis of NMOSD was established.

**Conclusion:**

High intracranial pressure headache; however rare, may be the first sign of the onset of the acute exacerbation phase of NMOSD.

Neuromyelitis optica spectrum disorder (NMOSD) is an autoimmune astrocytopathic disease affecting central nervous system (CNS). The disease has a variety of manifestations, both clinically and radiologically. On the one hand, none of the clinical symptoms are pathognomonic for NMOSD; and on the other hand, it includes a wide range of clinical symptoms (1, 2). NMOSD is mainly recognized by optic nerve and spinal cord involvement. Optic neuritis, longitudinal transverse myelitis and postrema area involvement, acute brain stem or diencephalic clinical syndrome and symptomatic involvement of cerebrum with compatible NMOSD-typical lesions in magnetic resonance imaging (MRI) are the main characteristic cores of this disease. Myelitis is revealed by weakness of extremities and longitudinally extensive involvement of spinal cord in MRI. Optic neuritis can present with visual obscuration in one or both eyes. Orbital MRI usually shows involvement of optic nerve. In addition, other parts of brain can be involved in NMOSD. Postrema area, diencephalon, and cerebral hemispheres are other important areas which could be damaged in this disorder (1, 3, 4). Aquaporin 4 antibody plays a central role in the pathogenesis of NMOSD. This antibody attaches to aquaporin 4 channels on foot processes of astrocytes and damages them (1, 3). The diagnosis of NMOSD has been based on combination of clinical, laboratory and imaging findings (1, 5). Immunosuppressive drugs are the most important treatment of NMOSD. However, some of these drugs including azathioprin, cellcept and rituximab are available in Iran and are used extensively in the treatment of patients with NMOSD (3, 6).

In NMOSD, the cerebrospinal fluid (CSF) profile can also cover a wide range (7). There is no detailed information on CSF pressure in these patients, but it is usually normal. Although it seems that, headaches are less common in NMOSD, headache is considered as one of the primary manifestations of NMOSD or one of the signs of exacerbation (8). However, co-existence of NMOSD and headache with the raised ICP is rare, especially with concurrent onset. The following is a rare case of NMOSD who had complains of headache with the increased intracranial pressure (ICP) in acute exacerbation of disease.

## Case presentation

**Figure 1:**
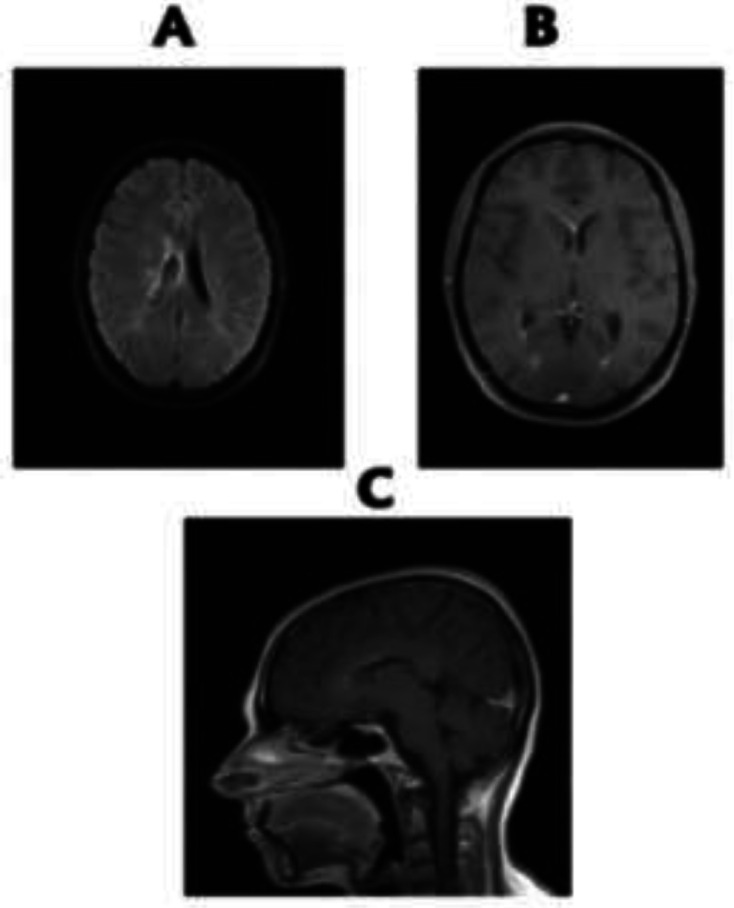
A. Brain MRI revealed periventricular lesions, B. periependymal enhancement and, C. linear enhancement of corpus callosum

**Figure 2:**
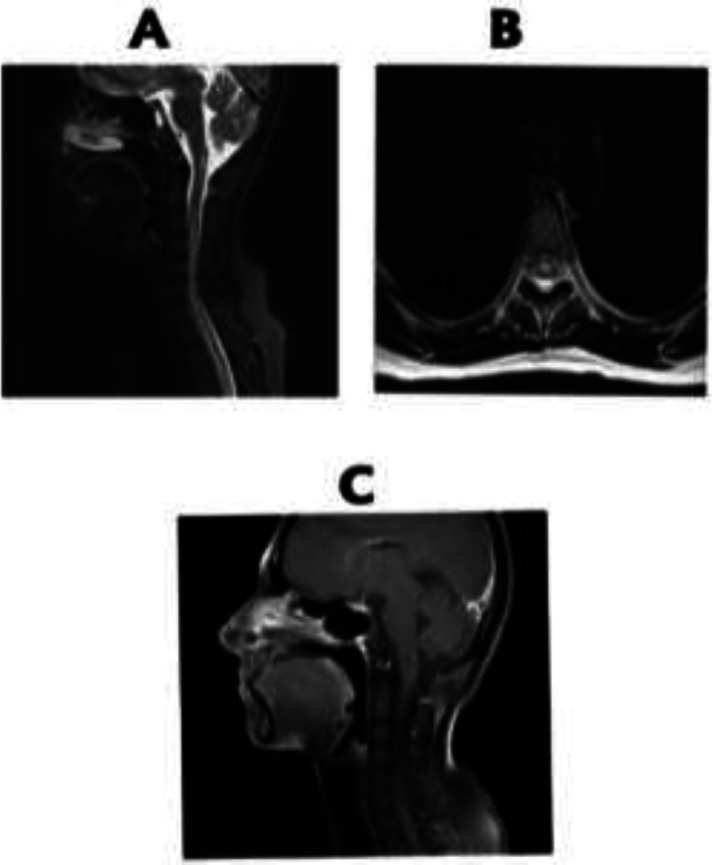
A. Cervical MRI revealed longitudinal extensive transverse myelitis (LETM) B. axial view showed central involvement of cervical cord. C. Cervical MRI with gadolinium revealed ring enhancement

A 30-year-old female patient presented to another center in September 2019 with primary manifestations of upper limb muscle spasms and was diagnosed as multiple sclerosis (MS). In November 2019, she was admitted in our center with complaints of headache and lower limbs paresis. The patient's headache was positional aggravated by lying down. Opthalmoscopy examination revealed bilateral papilledema. Paresis was progressive and within 4 days, had increased from force level 5 from 5, to 1 from 5, and also she had a sensory level belowT4, which all representing acute myelitis. Brain MRI revealed periventricular lesions, periependymal enhancement and linear enhancement of corpus callosum (Figure 1- A, B, C). Cervical MRI revealed longitudinal extensive transverse myelitis (LETM) with ring enhancement (Figure 2- A, B, C). 

Brain MR venography (MRV) was normal. Lumbar puncture (LP) was performed for her. Opening pressure (OP) was 42 cm H2O in the first LP, so we began tablet acetazolamide 250mg twice daily and tablet lasix 20mg daily for the patient. CSF profile was white blood cell (WBC) count 99 cells (85% lymphocytes, 15% neutrophil), glucose 45, protein 55, and red blood cell (RBC) count 10. The patient's CSF samples were sent for rule out of tuberculosis, brucellosis, and Cryptococcus, which all of them were negative and also all other vascular, autoimmune diseases, and malignancies were considered and ruled out. The second patient's LP also had OP 27 cm H2O. Due to severe presentation of disease, we also began the plasmapheresis and corticosteroid simultaneously (9). According to the clinical evidences and imaging, the patient's positive anti- aquaporin antibody (AQP4-IGg), the diagnosis of NMOSD was established in terms of the revised 2015 criteria, and rituximab was then ordered for her. After foregoing treatments, the patient's headache gradually improved, muscle force returned to 4/5, and the patient was discharged after 23 days. No other disease was found in the studies that explained the patient's headache.

## Discussion

Regarding our review, only two other NMOSD cases with high ICP have been reported so far. Table 1 compares the characteristics of these three cases (10, 11). The CSF profile of NMOSD patients can have different characteristics at different stages of the disease. Based on the previous studies including Jurius et al.'s study of 211 CSF samples of NMOSD patients, some patients had a normal CSF in remission phase, whereas the patients in the active phase of the disease have a variety of features including pleocytosis, the increased protein, and total Albumin and lactate correlated with the activity and length of the spinal cord lesions (7). CSF profile of our case also corresponds to the above description, indicating that, the disease is in the recurrent phase. 

High ICP headaches can occur in the context of concomitant diseases (including chronic meningitis, sarcoidosis, cerebral venous thrombosis, etc.) or secondary to medications like corticosteroids (12). Therefore, all these secondary causes should be considered. In this case, all possible causes have been ruled out by examinations, LP, and MRI, and the onset of headache was prior to any corticosteroid or immunosuppressant administration. Neuroinflammation and aquaporin channel dysfunction appear to have a stronger role compared to other causes of high ICP headache in NMOSD. Aquaporin channels are present as regulators of water homeostasis throughout the body. Also, aquaporin-4 channels are predominantly present in the brain. In NMOSD, antibodies are produced against these channels and their function is impaired, which is exacerbated by impaired permeability of the blood brain barrier and reduces the resolution of neuroinflammation in these patients (13). Based on a study conducted by Wang et al. on the CSF sample of NMOSD and MS patients and comparing the resolution of inflammation in them, it seems that, the presence of AQP4-IGg in CSF may delay the recovery of CNS inflammation and disease activity in these patients (14). We postulated that, this mechanism and its associated inflammation might have a role in the occurrence of high ICP in NMOSD. 

**Table1:** NMOSD associated to the case reports with the High intracranial pressure headache

**Keefe (1957)**	**Viswanathan et al (2017)**	**Our case**	
13	17	30	Age of onset
female	Male	female	Sex
Co-incidence	3 years	Co-incidence	Headache-NMOSD onset interval
sudden onset of severe frontal headaches associated with blurred vision	headaches, tinnitus and diploplia on lateral gaze due to a 6th nerve palsy	headache and lower limbs paresis and paresthesia below level T4	Clinical symptoms when admitted
Not available	Normal	Brain and cervical involvement	MRI features
Opening pressure 190 mm. of H2O, WBC count 99 cells/cu. mm. (86% lymphocytes, 14% polymorphonuclear cells), sugar 70 mg/100 cc, total protein 30.7 mg/100 cc, , and culture negative.	Opening pressures of 30 cm of H2O with normal CSF	Opening pressure of 42 Cm of H2O WBC count 99 cells/cu. (85% lymphocytes, 15% neutrophil), glucose 45, protein 55, RBC count 10	CSF features
Not available	Positive	Positive	AQP4-IgG serology

In conclusion, according to this case, high ICP headache, although it is rare, may be the first sign of the onset of the acute exacerbation phase of NMO. Keeping this in mind can help in more quickly diagnosing the disease, and to initiate treatment faster to prevent further complications.
